# Metabolic Engineering of *Corynebacterium glutamicum* for the Production of Flavonoids and Stilbenoids

**DOI:** 10.3390/molecules29102252

**Published:** 2024-05-10

**Authors:** Luan Luong Chu, Chau T. Bang Tran, Duyen T. Kieu Pham, Hoa T. An Nguyen, Mi Ha Nguyen, Nhung Mai Pham, Anh T. Van Nguyen, Dung T. Phan, Ha Minh Do, Quang Huy Nguyen

**Affiliations:** 1 Faculty of Biotechnology, Chemistry and Environmental Engineering, Phenikaa University, Hanoi 12116, Vietnam; 2 Faculty of Biology, University of Science, Vietnam National University, Hanoi (VNU), 334 Nguyen Trai, Thanh Xuan, Hanoi 10000, Vietnamhuy_nq@hus.edu.vn (Q.H.N.); 3 National Key Laboratory of Enzyme and Protein Technology, University of Science, Vietnam National University, Hanoi (VNU), 334 Nguyen Trai, Thanh Xuan, Hanoi 10000, Vietnam

**Keywords:** *Corynebacterium glutamicum*, metabolic engineering, flavonoid, stilbenoid, secondary metabolites

## Abstract

Flavonoids and stilbenoids, crucial secondary metabolites abundant in plants and fungi, display diverse biological and pharmaceutical activities, including potent antioxidant, anti-inflammatory, and antimicrobial effects. However, conventional production methods, such as chemical synthesis and plant extraction, face challenges in sustainability and yield. Hence, there is a notable shift towards biological production using microorganisms like *Escherichia coli* and yeast. Yet, the drawbacks of using *E. coli* and yeast as hosts for these compounds persist. For instance, yeast’s complex glycosylation profile can lead to intricate protein production scenarios, including hyperglycosylation issues. Consequently, *Corynebacterium glutamicum* emerges as a promising alternative, given its adaptability and recent advances in metabolic engineering. Although extensively used in biotechnological applications, the potential production of flavonoid and stilbenoid in engineered *C. glutamicum* remains largely untapped compared to *E. coli*. This review explores the potential of metabolic engineering in *C. glutamicum* for biosynthesis, highlighting its versatility as a cell factory and assessing optimization strategies for these pathways. Additionally, various metabolic engineering methods, including genomic editing and biosensors, and cofactor regeneration are evaluated, with a focus on *C. glutamicum.* Through comprehensive discussion, the review offers insights into future perspectives in production, aiding researchers and industry professionals in the field.

## 1. Introduction

**Figure 1:**
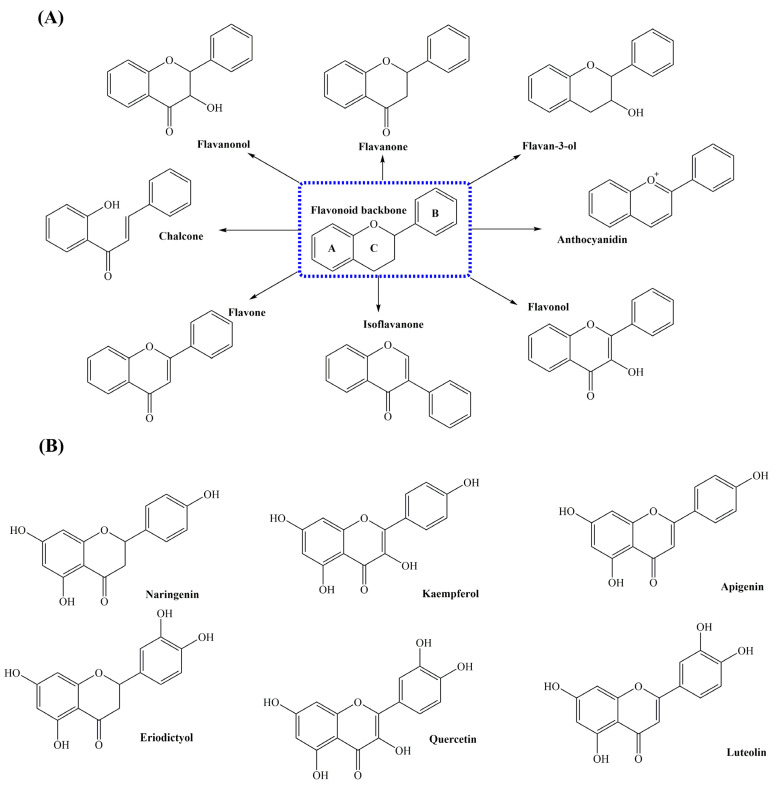
Basic structure of flavonoids. (**A**) The chemical structure with two phenyl rings (designated as rings A and B) connected by a heterocyclic ring (ring C) and classification of flavonoid. (**B**) The chemical structure of synthetic flavonoids in this study.

Flavonoids and stilbenoids represent vital classes of secondary metabolites renowned for their diverse biological and pharmaceutical activities. Flavonoids constitute a diverse and chemically intricate class of naturally occurring secondary metabolites that is primarily derived from plants [1]. They are characterized by a common structural framework, comprising a 15-carbon skeleton with two phenyl rings (designated as rings A and B) connected by a heterocyclic ring (ring C) (Figure 1). This unique structure forms the basis for the classification of flavonoids into several subclasses, each exhibiting specific variations in its chemical compositions. Flavonoids are categorized into distinct subclasses, including chalcones, flavanones, flavones, isoflavones, flavonols, flavanonols, flavanols, and anthocyanins (Figure 1) [2]. On the other hand, stilbenoids, characterized by a core structure consisting of two phenyl rings connected by a methylene bridge, represent a diverse group of polyphenolic compounds with significant pharmacological potential. This structural motif serves as the foundation for a variety of stilbenoid subclasses, including stilbenes, oligostilbenes, bibenzyls, bisbibenzyls, and phenanthrenes, each distinguished by unique arrangements of phenyl rings and substitution patterns [3].

**Figure 2:**
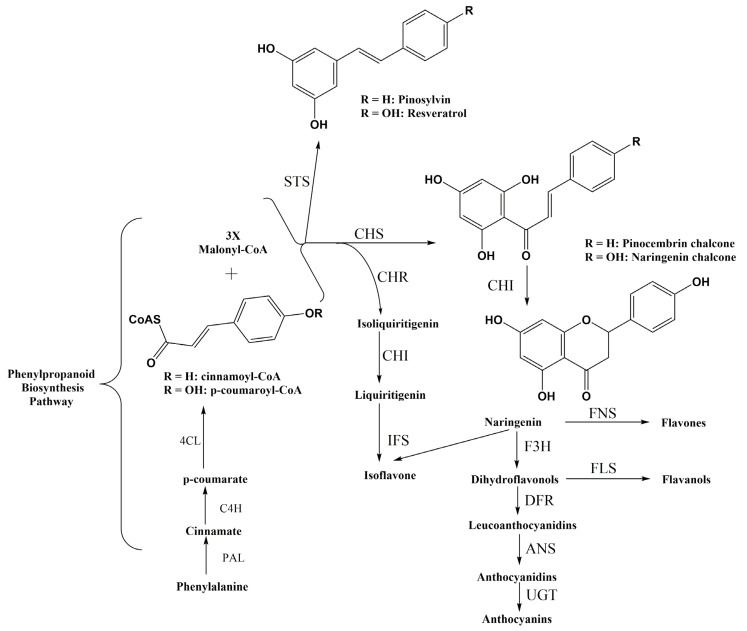
Schematic overview of the flavonoid biosynthesis pathway in plants. 4CL, 4-coumarate: CoA ligase; ANS, anthocyanidin synthase; C4H, cinnamate 4-hydroxylase; CHI, chalcone isomerase; CHR, chalcone reductase; CHS, chalcone synthase; DFR, dihydroflavonol 4-reductase; F3H, flavanone 3-hydroxylase; FNS, flavone synthase; FLS, flavonol synthase; IFS, isoflavone synthase; PAL, phenylalanine ammonia-lyase; STS, stilbene synthase; UGT, UDP-glucose glucosyltransferase.

Both flavonoids and stilbenoids play crucial roles in various physiological and biochemical functions within plants. For instance, flavonoids contribute to pigmentation for flower coloration, symbiotic nitrogen fixation, and protection against ultraviolet (UV) radiation. Beyond their significance in plants, flavonoids and stilbenoids exhibit notable physiological and biochemical effects in mammals, such as antioxidant activity, anti-inflammatory [4], antibacterial [5], and anticancer properties [6]. Their utilization spans across diverse industries, notably in food, where their antioxidant properties contribute to the preservation of food products [7]. In the nutraceutical sector, flavonoids play a vital role in promoting health and well-being, with specific compounds like apigenin demonstrating efficacy in mitigating metabolic syndrome (Figure 2) [8]. Despite the intrinsic value of flavonoids and stilbenoids, their purity remains contingent on environmental factors, presenting challenges in the manufacturing process.

Historically, flavonoid and stilbenoid production predominantly relied on conventional methods, chemical synthesis, and plant extraction, each beset with inherent challenges. The chemical synthesis approach is encumbered using hazardous materials, intricate processing conditions, and limited yield [9]. Simultaneously, plant extraction faces hurdles related to accessibility and supply chain logistics, owing to the scarcity of raw materials, low abundance in plants, and an overall limited product yield [10]. In response to these constraints, the industry has undergone a transformative shift towards sustainable and environmentally friendly alternatives. A notable alternative has emerged through the biological production of these two compounds, utilizing microorganisms such as *E. coli* and yeasts [11]. Until now, *E. coli* and yeasts have been the most common hosts for the production of flavonoids and stilbenoids. While engineered *S. cerevisiae* could obtain the highest naringenin with 648.63 mg/L by exogenously feeding *p*-coumaric acid [12], *E. coli* showed the highest isoorientin from luteolin, with 3829 mg/L [13]. Engineered *E. coli* and *S. cerevisiae* introduce the potential for large-scale production, harnessing renewable biomass sources like glucose and xylose. While this biological avenue mitigates certain challenges associated with chemical methods, it introduces new drawbacks specific to *E. coli* or yeast as production hosts [14,15]. In addressing these challenges and seeking to optimize flavonoid and stilbenoid biosynthesis, recent strides in metabolic engineering have spurred the exploration of alternative microbial hosts, with particular attention paid to *Corynebacterium*. The literature underscores the need for innovative microbial hosts to enhance efficiency and sustainability in flavonoid and stilbenoid production [16,17]. Notably, *Corynebacterium* has emerged as a promising candidate due to its unique characteristics and adaptability to metabolic engineering strategies [18]. Indeed, recent developments in multiomics methods, synthetic biology techniques, and metabolic engineering approaches have facilitated the transformation of recombinant *C. glutamicum* into a flexible microbial cell factory for generating high-value platform chemicals and polymers, using diverse sugars derived from biomass [19,20]. *C. glutamicum* has also been engineered for anthocyanin production. Anthocyanins are valuable flavonoids that have diverse applications in food processing, cosmetic production, and nutraceutical manufacturing [21]. This study opens up a greater possibility of *C. glutamicum* as a host microbe for the biosynthesis of useful and value-added natural compounds [22,23]. This literature review critically examines the intricacies of metabolic engineering strategies within microorganisms, providing an in-depth focus on the utilization of *Corynebacterium*. By comprehensively evaluating the strengths and limitations of *C. glutamicum* hosts, this review aims to furnish a nuanced understanding of the dynamic landscape in metabolic engineering for flavonoid and stilbenoid synthesis.

## 2. Flavonoids and Its Biological Activities

Flavonoids are found in fruits, vegetables, grains, bark, roots, flowers, tea, and wine. More than 8000 compounds with a flavonoid structure have been identified, and about 6000 of them were found to contribute to the color pigments of flowers, fruits, and leaves. Flavonoids are polyphenolic compounds and are a large family of plant secondary metabolites. Chemically, flavonoids possess a basic fifteen-carbon flavone structure: C_6_-C_3_-C_6_, consisting of two benzene rings (A-ring and B-ring), connected via a heterocyclic pyran ring (C-ring) (Figure 1). The antioxidant capacity of flavonoids is influenced by the position of the B-ring on the C-ring, and the position and number of hydroxyl groups on the catechol group of the B-ring. Based on the degree of central heterocyclic ring saturation, flavonoids are classified into eight subclasses: flavonols, flavones, isoflavones, anthocyanidins, chalcones, flavanones, flavan-3-ols, and flavanonols (Figure 1) [1,2]. This classification depends on several factors, such as the carbon of the heterocyclic ring to which the B-ring is attached, degree of unsaturation, degree of oxidation, degree of hydroxylation, glycosylation patterns, etc. In plants, flavonoids can be found in vacuoles of plant cells in the form of glycosides or linked to sugars.

Flavonoids are derived from the phenylpropanoid metabolic pathway, and their biosynthesis has long received intense research from plant biology (Figure 2) [24]. The first step in flavonoid biosynthesis is the conversion of phenylalanine to cinnamic acid by the enzyme phenylalanine ammonia-lyase (PAL). Cinnamic acid is then converted to *p*-coumaric acid by the enzyme cinnamate 4-hydroxylase (C4H). The next step involves the conversion of *p*-coumaric acid to naringenin chalcone. This reaction is catalyzed by the enzyme chalcone synthase (CHS). Naringenin chalcone is an important precursor in the biosynthesis of various flavonoids. Naringenin chalcone is further modified to produce different classes of flavonoids through a series of enzymatic reactions. For example, the enzyme chalcone isomerase (CHI) converts naringenin chalcone to naringenin, which is a precursor for flavanones. Flavanones can be further modified to produce flavones, flavonols, and isoflavones. Another branch of the flavonoid biosynthesis pathway involves the conversion of naringenin chalcone to dihydrokaempferol by the enzyme flavanone 3-hydroxylase (F3H). Dihydrokaempferol can be further converted to produce different classes of flavonoids, such as flavan-3-ols (catechins) and anthocyanins [24,25]. Anthocyanins and proanthocyanidins are synthesized along the general phenylpropanoid pathway by the flavonoid metabolon, a multienzyme complex of the cytochrome-P450 family, which is loosely attached to the cytoplasmic face of the endoplasmic reticulum (Figure 2). The biosynthesis of flavonoids is regulated by various factors, including environmental cues, hormonal signals, and transcription factors. This regulation ensures that flavonoid production is tightly controlled and can be modulated in response to different developmental and environmental conditions [25,26].

**Figure 3:**
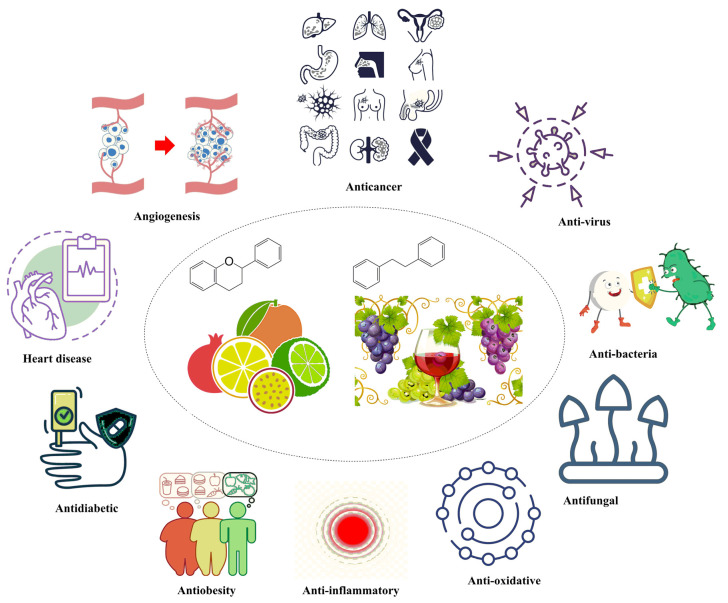
The biological activities of flavonoid and stilbenoid derivatives.

Flavonoids are proven to possess a wide spectrum of biochemical applications associated with health enhancing effects. The exemplary properties of those are antidiabetic, antiobesity, anti-inflammatory, antioxidative, antimicrobial, antiviral, differentiate, and apoptotic effects (Figure 3) [27]. Almost all groups of flavonoids are described to have antioxidant capacity. This property depends on configuration substitution and the number of hydroxyl groups. The mechanism of antioxidant action includes the suppression of reactive oxygen species (ROS) formation, scavenging ROS, and the upregulation and protection of antioxidant enzymes [28]. In plants, flavonoids are known as effective antimicrobial substances. Some of known flavonoids with potent antibacterial property are isoflavones, flavanones, chalcones, and apigenin [29]. Interestingly, many flavonoids have been recognized to have antiviral ability, and numerous reports on a variety of antiviral flavonoids have been published. Among those, some reports proved the significance of flavonoids in combating HIV (inhibits HIV-1 infection and replication, inhibits HIV-proteinase). Additionally, quercetin was found to be most potent against DENV-2 (dengue virus type-2). Moreover, flavonoids show potential inhibitory activity against coronaviruses and reduce the exacerbation of COVID-19 [30]. Furthermore, flavonoids have been found to exhibit anticancer properties by interfering with various stages of cancer development. They can inhibit the growth of cancer cells, induce apoptosis (programmed cell death), inhibit angiogenesis (formation of new blood vessels that supply tumors), and prevent metastasis (spread of cancer cells to other parts of the body). Flavonoids have shown potential in the prevention and treatment of various types of cancers, including breast, lung, colon, and prostate cancers [5]. Moreover, flavonoids have been shown to have beneficial effects on the cardiovascular system. They can improve blood vessel function, reduce inflammation in blood vessels, inhibit platelet aggregation (clumping of blood platelets), lower blood pressure, and enhance the production of nitric oxide, which helps relax blood vessels. These effects contribute to the prevention and management of cardiovascular diseases, including hypertension, atherosclerosis, and heart disease (Figure 3) [31].

## 3. Stilbenoids and Their Biological Activities

Stilbenoids are primarily found in plants, although they can also be present in certain fruits and fungi. Some well-known plant sources of stilbenoids include grapes, berries, peanuts, and Japanese knotweed. Certain fungi, such as *Aspergillus* species and some mushrooms, also produce stilbenoids as part of their defense mechanisms. Over 1000 compounds within this group have been identified, a significant increase from the approximately 100 compounds listed in 1980 and the around 300 compounds identified in 1995 [3]. Stilbenoids, classified as a subclass of polyphenolic compounds, derive their distinctive characteristics from a unique 1,2-diphenylethylene backbone, which entails a double bond linking two phenyl rings. This structural motif is pivotal in dictating the chemical interactions and functional properties of stilbenoids. The presence of the double bond confers rigidity to the molecular structure, influencing the spatial arrangement of functional groups and the overall reactivity of the compound. Additionally, the conjugated system formed by the double bond enhances the electron delocalization within the molecule, contributing to its antioxidant and radical scavenging capabilities [32]. 

The structural feature of stilbenoid imparts unique properties to stilbenoids, contributing to their diverse biological activities and pharmaceutical potential (Figure 3). For instance, stilbenes, the most common subclass of stilbenoids, typically contain hydroxyl groups at specific positions along the phenyl rings. These groups contribute to their antioxidant and anti-inflammatory properties [33]. Resveratrol, the most renowned member of this group, is found in the roots of *Polygonum cuspidatum* and various *Vitis* species. This compound serves as a phytotoxin synthesized by multiple plants in reaction to infection or stress, gaining recognition for its cardioprotective properties often associated with red wine consumption [34]. Epsilon-viniferin, a dimeric stilbenoid belonging to the oligostilbenes subclass, demonstrates broad-spectrum antimicrobial activity against bacteria and fungi, suggesting its potential as a natural antimicrobial agent. Similarly, marchantin A, a bisbibenzyl compound derived from liverworts, displays significant antimicrobial efficacy against various microorganisms [35]. In addition to antimicrobial effects, stilbenoids also possess notable antitumor activity. Riccardin D, a member of the bisbibenzyls subclass, shows promise in cancer therapy by inhibiting cancer cell proliferation and inducing apoptosis through targeting key signaling pathways. Moreover, certain stilbenoids, such as phylligenin, a bibenzyl derivative, exhibit anti-inflammatory properties by inhibiting the production of inflammatory mediators and cytokines [36]. Additionally, epidithiodioxopiperazines (ETPs), a subclass of phenanthrenes produced by fungi, demonstrate potent antifungal activity against pathogenic fungi like *Candida albicans* and *Aspergillus fumigatus* [37].

**Figure 4:**
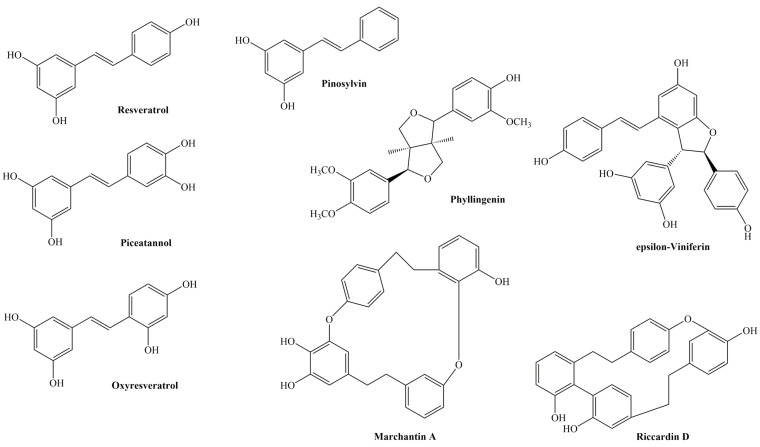
Chemical structures of stilbenes and related stilbenoids in this study.

Like flavonoids, the biosynthesis of stilbenoids is intricately regulated by a combination of environmental cues, hormonal signals, and transcription factors. This intricate regulation ensures that stilbene production remains tightly controlled and can be modulated in response to varying developmental and environmental conditions. The synthesis pathway of stilbenoids initiates within the phenylpropanoid pathway, a pivotal metabolic route in plants crucial to produce various secondary metabolites [38]. Phenylalanine, an amino acid, serves as the precursor molecule for stilbenes, the fundamental building blocks of stilbenoids. This biosynthetic process begins with the enzymatic action of PAL, which converts phenylalanine to cinnamic acid, marking the initial step in stilbene biosynthesis. Subsequently, cinnamic acid undergoes further modifications to generate various stilbenes. Stilbene biosynthesis is primarily facilitated by stilbene synthase (STS), a key enzyme that catalyzes the condensation of three molecules of malonyl-CoA with one molecule of cinnamoyl-CoA, leading to the formation of resveratrol, a prominent stilbenoid compound. Resveratrol serves as a precursor for the biosynthesis of other stilbenoids, such as pterostilbene, oxyresveratrol, and viniferins (Figure 4) [32]. Environmental factors, including UV radiation, pathogen attacks, and abiotic stress, influence stilbene production, while cell signaling pathways, notably the jasmonic acid (JA) and salicylic acid (SA) pathways, regulate stilbene synthesis [39,40]. Insight into these natural mechanisms not only aids in understanding plant defense responses but also holds promise for the development of plant protection strategies and the enhancement of stilbene production for various commercial applications [41].

## 4. *Corynebacterium glutamicum* as a Novel Platform to Produce Flavonoids and Stilbenoids

*Corynebacterium glutamicum* is a Gram-positive, non-sporulating, and non-pathogenic bacterium which is used for high production of numerous amino acids annually, especially L-glutamate and L-lysine, in addition to L-tryptophan [42]. Although *C. glutamicum* grows quickly to high cell densities as a bacterial model *E. coli*, it can be easily propagated to a large scale with no autolysis. Moreover, *C. glutamicum* is Generally Recognized as Safe (GRAS) by the Food and Drug Administration (FDA) due to the fact that it does not produce endotoxins or undergo phage lysis [43]. Unlike other industrial microbes, such as *E. coli* and *B. subtilis*, *C. glutamicum* exhibits high tolerance to various toxic compounds. The reason for this is that it possesses a robust cell wall with a thick glycan core or a crystalline surface S-layer [44]. These features make *C. glutamicum* is an alternative microbe for the production of a range of commercial aromatic compounds.

Importantly, the whole genome sequence of *C. glutamicum* was published in 2003 as a fundamental understanding paving the way for a better knowledge and more approachable for genetic engineering [45]. According to a well-established understanding of *C. glutamicum* genomic, genetic elements (a wide range of low- and high-copy number shuttle vectors, promotors, selection markers, and reporter genes) have been developed, characterized, and modularized to express the target genes [42]. Moreover, along with classic molecular genetic manipulation techniques, the development of multiple omics approaches (transcriptome, proteome, metabolome, and fluxome) reveals molecular interactions and functioning of multi-molecular metabolic pathways in engineered *C. glutamicum*. In addition, computer simulations provide an efficient approach to predicting the metabolic pathway and optimum flux states in engineered strains [46]. These advantages allow for the metabolic engineering of *C. glutamicum* for the production of commercial compounds in industrial biotechnology. It is reported that the metabolic engineering of *C. glutamicum* could be obtained from over 70 different compounds, including flavonoids and stilbenoids [42].

Most kinds of wild-type *C. glutamicum* lack effective arabinose and xylose transporters for metabolic pathway; however, the engineered *C. glutamicum* could simultaneously consume various carbon substrates. Engineered *C. glutamicum* not only showed the ability to hydrolyze lignocellulosic biomasses but also exhibited the utilization of different sugars, including many hexoses and pentose. This characteristic of engineered *C. glutamicum* allows for the biosynthesis of a range of commercial products with renewable resources as eco-friendly feedstock for fermentation [47]. Interestingly, it is found that *C. glutamicum* is able to produce *p*-coumaric acid, ferulic acid, caffeic acid, and 3-(4-hydroxyphenyl)propionic acid, but not for cinnamic acid, through a degradation of phenylpropanoids, which are used as sole carbon and energy source. The degradation of phenylpropanoids was reported involving the Phd pathway. Furthermore, there was the absence of the genes *cg0341, cg0344, cg0345,* and *cg0347*, which were essential for phenylpropanoid degradation. These genes play pivotal roles in the breakdown of phenylpropanoids within the metabolic pathway. Although the deletion of the genes for the phenylpropanoid transporter PhdT (*cg0340*) resulted in a decline in the growth rate with phenylpropanoid, its appearance was not significant for the development with these compounds [48]. On the other hand, *C. glutamicum* failed to grow on cinnamic acid as sole carbon and energy source due to the low interaction of cinnamic acid with the repressor protein affecting the expression of the genes in the catabolism. In addition, it is confirmed that *C. glutamicum* is unable to utilize phenylpropanoids as a sole carbon and energy source when being deleted from three gene clusters, *cg0344*-47, *cg2625-40* (genes coding for enzymes of β-ketoadipate pathway), and *cg1226* (genes *pobA*), which did not contain any regulated or transported genes. This feature is one of the greatest advantages of *C. glutamicum* in regard to the production of flavonoids because it is demonstrated that phenylpropanoids represent a significant precursor for the biosynthesis of secondary metabolites, including flavonoids and stilbenes [49]. Noticeably, when investigated after given either quinate or shikimate as the only carbon source, *C. glutamicum* developed well. The shikimate pathway in *C. glutamicum* is primarily controlled by the enzymes engaged in feedback-inhibition at the transcriptional stage. The catabolic pathway for quinate and shikimate and the anabolic shikimate pathway to produce aromatic amino acids overlap during the 3-dehydroshikimate stage. A catabolic pathway occurs when the level of shikimate pathway’s metabolites increases, resulting in the production of hydroxybenzoic acid, protocatechuate from 3-dehydroshikimate. Protocatechuate then degraded to succinyl-CoA and acetyl-CoA through the β-ketoadipate pathway in as energy [50]. In addition to using quinate or shikimate as carbon source, *C. glutamicum* also prevents the unwanted overproduction of aromatic amino acids from using carbon and energy. Due to its many advantages, *C. glutamicum* represents an interesting platform for production of various flavonoids and stilbenoids.

## 5. Metabolic Engineering of *Corynebacterium glutamicum* Cell Factories for Biosynthesis of Flavonoids and Stilbenoids

The metabolic engineering of *C. glutamicum* combines several individual engineering strategies for the highest productivity of flavonoids and stilbenoids. Due to the fact that most of the chemical structures of these compounds are derivatives of phenylpropanoids, such as resveratrol, naringenin, eriodictyol, and pinocembrin, the major of the research in engineered *C. glutamicum* focuses on reconstructing flavanones and the resveratrol biosynthetic pathway. In this section, we introduce three major metabolic engineering strategies for the production of flavonoids and stilbene in engineered *C. glutamicum,* including the expression of heterologous genes via an artificial pathway, precursor pools by overexpression, and the deletion of competing pathways.

### 5.1. Establishing an Artificial Pathway

**Figure 5:**
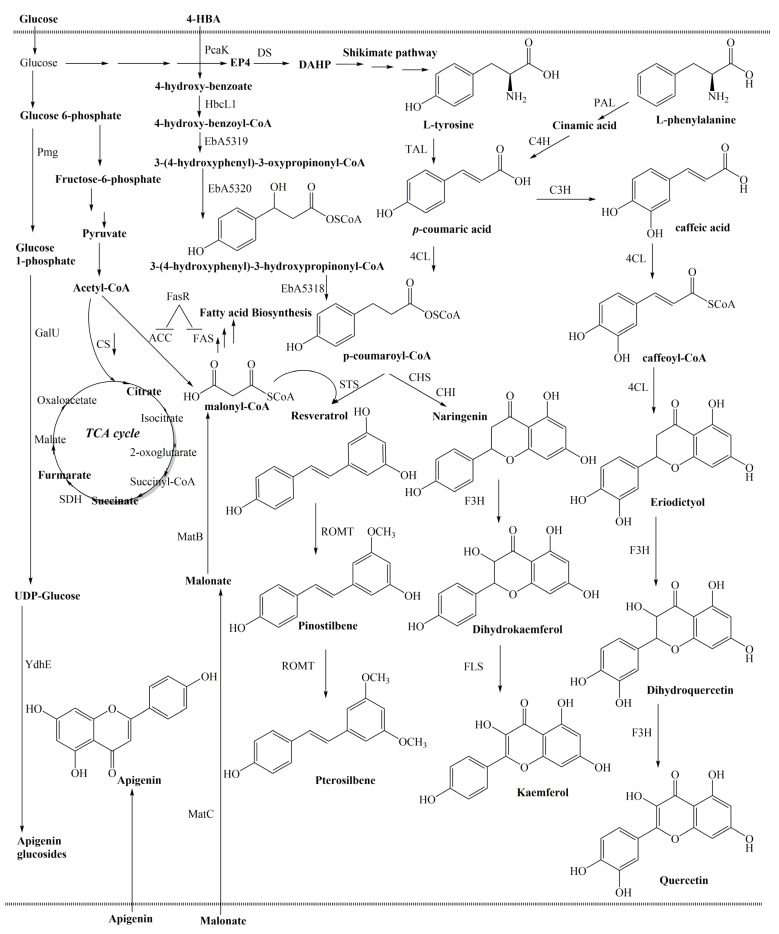
The proposed biosynthetic pathway for flavonoids production for engineered *C. glutamicium.* ACC, acetyl-CoA carboxylase; C4H, cinnamate-4-hydroxylase; HbcL1, EbA5368, 4-hydroxybenzoate: CoAligase; EbA5319, acyl-CoA: C-acetyltransferases; EbA5320, 3-hydroxyacyl-CoA dehydrogenase. galU1, UDP-D-glucose pyrophosphorylase; TAL, tyrosine ammonia-lyase; matB, malonyl-CoA synthetase; matC, malonyl-CoA de-carboxylase from *Rhizobium trifolii*; pmg, phosphoglucomutase; YdhE, a glycosyltransferase from *Bacillus lichenformis*.

Unlike flavonoid and stilbenoid biosynthesis in plants, an artificial pathway is required to be introduced into *C. glutamicum*. The pathway enzymes are then expressed and produced flavonoid and stilbenoids. The introduction of an artificial pathway into *C. glutamicum* is the most important part of metabolic engineering for flavonoid and stilbenes biosynthesis. Although various flavonoids and stilbenoids with different productivities could be obtained from various plants, our understanding of the biosynthesis pathway genes is limited due to a lack of genetic information from their natural hosts. Therefore, the expression of genes from plants into *C. glutamicum* is required for flavonoid and stilbenoid biosynthesis. For example, it is demonstrated that using *CHS* and *CHI* of *Petunia hybrida* (*PhCHS* and *PhCHI*) could produce naringenin and eriodictyol in engineered *C. glutamicum*. Furthermore, *STS* from peanut (*Arachis hypogaea*) (*AhSTS*) and *4CL* from parsley (*Petroselinum crispum*) (*Pc4CL*) were expressed for the production of pinosylvin, resveratrol, and piceatannol in engineered *C. glutamicum* (Figure 5) [48]. 

*C. glutamicum* can grow on phenylpropanoids such as *p*-coumaric acid, ferulic acid, caffeic acid, 4-hydroxybenzoic acid, and 3-(4-hydroxyphenyl) propionic acid [51,52]. Although the disadvantages are the precursor cost and toxicity of these intermediate, the feeding of phenylpropanoids into the culture medium resulted in increased flavonoid and stilbenoid production, without modulation of the biosynthetic pathway. For example, *p*-coumaric acid and caffeic acid were used as a precursor to synthesize kaempferol and quercetin in *C. glutamicum* expressing the genes *Pc4CL* and *PhCHS;* and *PhCHI, PhF3H, and PdFLS,* respectively [51]. Similarity, supplementation of *p*-coumaric acid into the culture medium of *C. glutamicum MB001DelAro^4^* resulted in the production of resveratrol (Table 1) [53]. Interestingly, a β-oxidative phenylpropanoid degradation pathway was identified for the synthesis of phenylpropanoid CoA-thioesters, starting from cheap benzoic acids in engineered *C. glutamicum*. 4-Hydroxybenzoate is imported into *C. glutamicum* DelAro^4^ pMKEx2_*stsAh*_*4clPc* pEKEx3_RevBeta via the endogenous transporter *PcaK*. Then, the conversion of 4-HBA to 4-hydroxybenzoyl-CoA is catalyzed by 4-hydroxybenzoate: CoAligase (*HbcL1, EbA5368*). In the next step, the formation of 3-(4- hydroxyphenyl)-3-oxopropionyl-CoA from 4-hydroxybenzoylCoA and acetyl-CoA is catalyzed by the β-ketothiolase (acyl-CoA: C-acetyltransferases; *EbA5319*). In the next stage, 3-hydroxyacyl-CoA dehydrogenase (*EbA5320*) is converted the 3-(4-hydroxyphenyl)-3-oxopropionyl-CoA to 3-(4-hydroxyphenyl)-3-hydroxypropionyl-CoA. Then, *p*-coumaroyl-CoA is formed by the conversion of 3-(4-hydroxyphenyl)-3-hydroxypropionyl-CoA to *p*-coumaroyl-CoA. Finally, *p*-coumaroyl-CoA and three molecules of malonyl-CoA are combined by the STS for the synthesis of resveratrol. Four genes, namely *EbA5368*, *EbA5319*, *EbA5320*, and *EbA5318*, were isolated from “*Aromatoleum aromaticum*” EbN1 under control of *Ptuf* (Figure 5) [42]. In another case, engineered *C. glutamicum* could produce anthocyanin C3G from precursor catechin [14]. Furthermore, *C. glutamic*um was engineered to allow for polyphenol production directly from aromatic amino acids supplementation in the culture medium. It is demonstrated that engineered *C. glutamicum* Nor2 C5 mu*fasOBCD1* PO6-*iolT1* ∆*pyc* could produce noreugenin in culture medium supplemented with casamino acids [54]. More recent research reported that the addition of tyrosine into the culture medium of engineered *C. glutamic*um could result in produced eriodictyol. This engineered *C. glutamicum* harbored *Pc4CL*, *PhCHS,* and *MsCHI*; *matB* (encoding malonyl-CoA synthetase) and *matC* (encoding malonyl-CoA decarboxylase) from *Rhizobium trifolii* (*RhmatBC*); and *hpaBC* (encoding 4-hydroxyphenylacetate 3-hydroxylase) genes from *E. coli* (*EchpaBC*) [55]. 

### 5.2. Enhancement of Intracellular Malonyl-CoA Level

The introduction of the heterologous pathway in bacteria is the most important part of metabolic engineering for flavonoid and stilbenoid biosynthesis. However, this approach might cause an unbalanced cellular metabolic flux. The competitive consumption of the precursor metabolite between the endogenous and heterologous pathways not only results in a reduction of target compound productivity but also affects cell growth. Therefore, balancing and increasing these factors would facilitate the enhancement of flavonoid and stilbenoid production in engineered *C. glutamicum.* According to the flavonoid and stilbenoid biosynthetic pathway, malonyl-CoA is one of the most important precursors for the formation of naringenin—a central intermediate for conversion to flavonoid in the Phd pathway (Figure 2) [56]. Although endogenous malonyl-CoA of *C. glutamicum* is synthesized from acetyl-CoA by acetyl-CoA carboxylase (ACC), the level of malonyl-CoA in cytosol is low. Therefore, the engineering in *C. glutamicum* is required to increase product titers of malonyl-CoA. Several approaches have been applied to improve malonyl-CoA levels. 

In the traditional method, the addition of cerulenin is known to inhibit fatty acid synthesis, which leads to improvement of the intracellular malonyl-CoA as a side effect [59]. For example, the titer of naringenin was increased 18% from 22 to 26 mg/L when 25 uM cerulenin was present in the medium culture of the strain *C. glutamicum Nar1* pEKEx3_*accBC*_*accD1*. In another way, it was not always successful; however, overexpression of gene coding for ACC subunits from native microorganisms and various sources is one of the most efficient ways to increase the intracellular malonyl-CoA availability during plant polyphenol. Interestingly, episomal overexpression of native ACC from *C. glutamicum* resulted in increased malonyl-CoA-dependent naringenin biosynthesis in *C. glutamicum* itself. The reason is because the ACC of *C. glutamicum* requires only two subunits (*accB1* and *accD1*), instead of four subunits for catalytic activity, as in *E. coli* [56]. Four subunits of ACC complex in *E. coli* include biotin carboxyl carrier protein (*BCCP*; *accB*), biotin carboxylase (*accC*), and two proteins (*accA* and *accD*) catalyzing the carboxyltransferase partial reaction [60]. In recent years, episomal overexpression of *accBC* (*cg0802*) and *accD1* (*cg0812*) was replaced by the TetR-type transcriptional repressor *FasR*; it effectively increases malonyl-CoA availability and accumulation of the flavonoid naringenin. *FasR* not only repressed the expression of *fas-IA* (*cg2743*) and *fas-IB* (*cg0957*) coding for the two fatty acid synthases, but also the deletion of FasR lead to the deregulation of the expression of *accBC* (*cg0802*) and *accD1* (*cg0812*). Therefore, *FasR* has a significant role in improving malonyl-CoA availability in cytosol of *C. glutamicum* (Figure 5) [56].

Glucose was used as carbon source for noreugenin synthesis in engineered *C. glutamicum*. Therefore, intracellular malonyl-CoA pool could be increased when the level of glucose into the cytosol of *C. glutamicum* is high. It is reported that the uptake of both D-xylose and D-glucose in *C. glutamicum* was increased through the *IolR*-mediated repression of the *iolT1* gene encoding the glucose/myo-inositol permease IolT1 [61]. *C. glutamicum* Nor2 C5 *mufasO_BCD1_ P_O6_-iolT1* has only a minor positive effect on noreugenin synthesis (4.40 mg/L in comparison with 4.13 mg/L of *C. glutamicum* Nor2 C5 mufasO*_BCD1_*); it also provides an alternative method for improving other natural products on *C. glutamicum* [54]. In another case, naringenin biosynthesis could be increased through a heterogeneous pathway from malonate to malonyl-CoA. This malonyl-CoA pathway has two genes, *matC,* encoding the malonate transporter, and *matB,* encoding the the malonyl-CoA synthase from *Rhizobium leguminosarum*. Both genes were expressed under the control of the *tac* promoter in the presence of sodium malonate. Interestingly, the highest titer increased by 83% in comparison with the strain without *matBC* (Figure 5) [55].

### 5.3. Deletion and Downregulation of Competitive Pathway

Since fatty acid synthesis and the tricarboxylic acid cycle (TCA cycle) are known as a metabolic pathway for consuming malonyl-CoA, reducing flux into the TCA cycle is also alternative approach to improving malonyl-CoA pool in *C. glutamicum.* One of the strategies in this case is the oxidation of acetyl-CoA in the TCA cycle. Acetyl-CoA is converted to malonyl-CoA by enzyme acetyl-CoA carboxylase; however, acetyl-CoA and oxaloacetate are considered to citrate by the citrate synthase (CS) in TCA cycles [62]. Noticeably, the downregulation of *gltA* encoding CS was carried out by exchanging its native promoter for a weaker promoter. In this case, the production of naringenin was increased 10-fold to 19 mg/L with 10% residual CS activity and 70% of the growth rate in comparison with the reference strain. According to the research in engineered *E. coli*, the deletion of the *sdhCAB* (cg0445-47) operon encoding the succinate dehydrogenase complex (*SDH*) was carried out to reduce growth rate and biomass in *C. glutamicum* (Figure 5). As a result of this mutation, C. glutamicum *Nar1 ΔsdhCAB* accumulated twice as much naringenin (4 mg/L) during the 20% reduction in the growth rate and 50% reduction in final biomass [54].

### 5.4. Improving Intracellular UDP-Sugar Level for Biosynthesis of Diversified Flavonoids

Since the backbone of flavonoid is based on a 15-carbon skeleton with two phenyl rings connected by a heterocyclic ring (ring C), the biosynthetic pathway of diversified flavonoids requires various types of functional enzymes. The modified derivatives of flavonoids are synthesized by methylation, hydroxylation, glycosylation, isoprenylation, and halogenation [63,64]. The diversified flavonoids showed improved bioavailability compared with flavonoid aglycone. For example, piceatannol is a hydroxylated compound from resveratrol, and it showed the highest cancer chemo-preventive activities [65]. Moreover, the prenylation of stilbenes showed good cancer preventive activities [66]. The physicochemical and biochemical properties of flavonoids could modulate through glycosylation reactions. It usually is more soluble, more stable, and more functional compared to their aglycones [67]. Furthermore, some research has demonstrated that the glycosylation of flavonoids had shown higher antioxidant activities; stronger hepato-, cardio-, and vasoprotectant behavior; and free radical scavengers than aglycone [68,69]. 

Although various types of diversified flavonoids have been synthesized in *E. coli* and *S. cerevisiae*, glycosylation of flavonoids has been reported as only modification of flavonoid in engineered *C. glutamicum* [57,58]. Glycosylation is catalyzed by glycosyltransferase, which transfers sugar molecules into the oxygen, carbon, nitrogen, and sulfur atoms of aglycon. O-glucosides and C-glucosides have been reported in numerous studies. For example, isoflavone 7-O-glucosyltransferase (IFS7GT) has been identified as a pivotal enzyme in this context, catalyzing the glycosylation of isoflavonoids [70]. Anthocyanidin 3-O-glucosyltransferase (A3OGT) serves as a potential alternative, focusing on the glycosylation of anthocyanidins and, thus, diversifying the pool of glycosylated flavonoid derivatives [71]. However, very few studies on N-glucosides have been reported. Recently, the N-glycosyltransferase activity of YdhE from *Bacillus lichenformis* has been shown in *C. glutamicum* [72]. One of the disadvantages of glycosylation in *C. glutamicum* is low availability of intracellular NDP-sugar, which is used as an essential donor for glycosyltransferase to the substrate. Therefore, increasing the intracellular UDP-glucose level has a good effect on the biosynthesis of flavonoid glucosides. The heterologous expression of the UDP-glucose pathway and blocking of the competitive UDP-glucose consumption pathways genes are general approaches to improving intracellular amount of UDP-glucose. The two UDP-glucose biosynthesis pathways from *E. coli* were introduced into *C. glutamicum* for the anthocyanin. The first pathway includes three genes, namely *cmk*, *ndk*, and *galU*, responding to the metabolic pathway of UDP-glucose biosynthesis from orotic acid. It is demonstrated that introduction of these genes into engineered *C. glutamicum* did not increase C3G titer. The reason is that the heterologous genes from *E. coli* were not able to coordinate with the native UDP-glucose biosynthesis for UDP-glucose accumulation. However, the supply of UDP-glucose consists of genes *pgm* (*cg2800*) and *galU1* (*cg1004*) resulted in a 4.2-fold higher C3G production in engineered *C. glutamicum* (reaching 31.8 mg/L) [20].

## 6. Future Perspectives and Conclusions

Although *E. coli* has been used along with yeasts for production of various bioactive compounds, *C. glutamicum* has increased the number of biotechnological processes for amino acids and organic acids production [73]. A lot of biotechnological strategies have successfully applied to *E. coli* for the efficient production of flavonoids and stilbenoids, including cofactor regeneration and optimization, protein engineering, genomic editing, biosensor-assisted evolution, and the optimization of fermentable condition [74,75,76,77]. Along with the wealth of knowledge on central physiology, genetic information, and carbon metabolism of *C. glutamicum*, these methods are expected to successfully increase the production efficiency of flavonoids and stilbenoids.

A critical avenue of exploration in *Corynebacterium* metabolic engineering focuses on the meticulous optimization of cofactor regeneration systems, with particular emphasis on vital cofactors such as Nicotinamide Adenine Dinucleotide Phosphate (NADPH), Nicotinamide Adenine Dinucleotide (NADH), Flavin Adenine Dinucleotide (FAD), and others. This strategic approach aims to enhance the availability of these cofactors, thereby fortifying the efficiency of flavonoid and stilbenoid biosynthetic pathways within the host organism [78]. The biosynthesis of flavonoid and stilbenoid involves intricate enzymatic reactions, many of which are dependent on specific cofactors. NADPH, as a primary reducing agent, plays a crucial role in facilitating the redox reactions essential for the conversion of precursors into flavonoid and stilbenoid. Simultaneously, NADH and FAD contribute to the redox balance and electron transfer processes, influencing various enzymatic steps in flavonoid pathways [79]. The optimization of cofactor regeneration systems is not merely a technical detail; it is a strategic intervention designed to address the diverse redox demands associated with flavonoid biosynthesis. By fine-tuning the cellular machinery responsible for regenerating NADPH, NADH, FAD, and other cofactors, researchers aim to create an environment conducive to sustained and efficient flavonoid production in *Corynebacterium*. This multifaceted approach recognizes the importance of balancing and optimizing a spectrum of cofactors, each with its unique role in the intricate network of biochemical reactions leading to flavonoid and stilbenoid synthesis.

Advancing enzyme expression represents a crucial strategy aimed at elevating the intracellular concentrations of key enzymes within the flavonoid and stilbenoid biosynthetic pathway. In the pursuit of enhancing enzyme activity, one of the key principles lies in improving the catalytic efficiency of enzymes within the flavonoid and stilbenoid biosynthetic pathway. This involves employing innovative strategies, such as directed evolution, computational design, and protein engineering, to optimize enzyme kinetics. The purpose of this method is to accelerate the conversion of specific precursor molecules, contributing to increased overall flavonoid and stilbenoid production. For example, CHS is known as the most important enzyme responsible for flavonoid production. The engineering of CHS results in increasing the efficiency of chalcones and downstream products in microorganisms [80]. In addition, unlocking substrate-flexible enzymes is a forward-looking strategy focused on enhancing the adaptability of enzymes to a diverse range of precursor molecules. This involves exploring enzymes with broad substrate specificity, thereby expanding the biosynthetic possibilities and structural diversity of flavonoid and stilbenoid. Research has shown that the sugar-donor specificity of UDP sugar: Glycosyltransferases (UGTs), which are responsible for conjugating flavonoids with various sugar moieties, can evolve locally in specific plant lineages. For example, an Arg residue in the PSPG box of Lamiales F7GATs was found to be critical for the specific recognition of UDP-glucuronic acid (UDPGA). Substituting this Arg with Trp was sufficient to convert the sugar-donor specificity of the Lamiales F7GATs from UDPGA to UDP-glucose [81]. In another study, three novel UDP-glycosyltransferases (PlUGT4, PlUGT15, and PlUGT57) were identified in *Pueraria lobata*. Among these, PlUGT4 displayed the utilization of a broad range of sugar acceptors [82]. These findings suggest that it might be possible to engineer flavonoid 5’-O-glucosyltransferase (F5’OGT) with broad substrate specificity by manipulating the amino acid residues involved in sugar donor recognition. As an alternative, flavonoid 3’-O-glucosyltransferase (F3’OGT) could offer a similar potential, enabling the glycosylation of a diverse array of precursor molecules and further enriching the structural diversity of glycosylated flavonoids. However, more research is needed to fully understand the molecular mechanisms underlying the substrate specificity of these enzymes. Pioneering synthetic enzyme scaffolds represents an avant-garde approach aimed at orchestrating the spatial arrangement of enzymes within the flavonoid biosynthetic pathway. The core principle involves creating tailored environments that optimize the interaction and collaboration between enzymes, promoting more efficient metabolic flux and pathway efficiency. By strategically organizing enzymes, researchers aim to enhance the overall productivity of flavonoid biosynthesis in *C. glutamicum*. Within this method, CHI and FLS emerge as central components of the synthetic enzyme scaffold. CHI is the second rate-limiting enzyme in the biosynthetic pathway of flavonoids, catalyzing the conversion of the bicyclic chalcone into tricyclic (2*S*)-flavanone and catalyzing the conversion of dihydroflavonols to flavonols [83]. By strategically positioning these enzymes in a synthetic scaffold, it is possible to facilitate the direct channeling of substrates from one enzyme to the next. This can prevent the diffusion of intermediates away from the enzyme surface, thereby increasing the overall rate of product formation. Moreover, such an arrangement can also minimize unwanted side reactions and improve the stability of the enzymes, further enhancing the efficiency of the biosynthetic pathway. In this innovative strategy, the envisioned outcome is a meticulously designed *C. glutamicum* strain with an optimized synthetic enzyme scaffold,

Genomic editing techniques offer unprecedented precision in manipulating microbial genomes, providing a powerful toolset for optimizing metabolic pathways and enhancing the production of valuable compounds, such as flavonoid and stilbenoid. In *C. glutamicum*, a bacterium widely used in industrial biotechnology, genomic editing holds immense promise for boosting flavonoid and stilbenoid yields through targeted modifications of key metabolic genes. This distinction is further exemplified in a study using the gene *zwf* which encodes glucose-6-phosphate dehydrogenase [84]. This enzyme plays a crucial role in the pentose phosphate pathway (PPP), facilitating the generation of NADPH, a vital cofactor in numerous biosynthetic reactions. The downregulation of *zwf* was proposed as a viable approach to redirect metabolic flux towards the production of erythrose-4-phosphate and sedoheptulose-7-phosphate, essential intermediates in aromatic amino acid, flavonoid, and stilbenoid biosynthesis. Another potential targeted gene regarding genomic editing is *pgi*, which encodes phosphoglucose isomerase. This enzyme catalyzes the interconversion of glucose-6-phosphate and fructose-6-phosphate, linking glycolysis to the pentose phosphate pathway. By modulating the activity or expression of *pgi,* researchers can manipulate carbon flux towards the pentose phosphate pathway, increasing the availability of precursor metabolites for flavonoid biosynthesis [85,86]. Generally, through genomic editing techniques, such as CRISPR-Cas9-mediated strategies, researchers aim to modulate NADPH availability, thereby influencing the flux of carbon towards flavonoid precursors. However, several challenges persist, including off-target effects, metabolic burden, and regulatory constraints. Addressing these challenges requires interdisciplinary approaches, encompassing computational modeling, synthetic biology, and systems biology.

Recent studies have illustrated the efficacy of genetically encoded biosensors in improving naringenin production. By dynamically regulating the expression of pathway enzymes in response to cellular conditions, these biosensors contribute to enhanced control over flavonoid biosynthesis. However, the success of such strategies hinges on the unique specifications of the biosensors, emphasizing the need for their reconstruction to achieve functional fine-tuning or improvement. Insights gained from previous research, particularly in hosts like *E. coli* or yeast, have provided valuable examples of successful biosensor implementation for metabolite tracking and pathway regulation [87,88,89]. These approaches enable the adaptation and optimization of biosensors to suit the intricacies of the host organism, particularly *C. glutamicum*, providing a foundation for tailored improvements in flavonoid production. An innovative strategy involves the assembly of well-optimized biosensors into genetic circuits to exert precise control over essential pathway enzymes. This approach not only makes flavonoid and stilbenoid production more sophisticated but also addresses the need for balancing enzyme expression levels. Translational control can be achieved using a ribosome binding site (RBS) library [90]. One of the newly developed biosensors is manonyl-CoA biosensor. Manonyl-CoA serves as an efficient platform for rapidly developing strains capable of producing valuable natural products. By precisely monitoring intracellular malonyl-CoA levels, this biosensor optimizes biosynthesis pathways. Unlike earlier biosensors limited to specific hosts, manonyl-CoA is applicable in three industrially important bacteria: *E. coli*, *Pseudomonas putida*, and *C. glutamicum*. Repurposing Type III polyketide synthase (*RppA*) as the biosensor allows for the easy identification of strains with enhanced malonyl-CoA accumulation. Through targeted gene knockdowns, engineered strains achieve impressive production levels of compounds like 6-methylsalicylic acid, aloesone, resveratrol, and naringenin [91].

The future of metabolic engineering for flavonoid and stilbenoid biosynthesis in *C. glutamicum* envisions a pivotal role for fermentation strategies. The core principle involves optimizing the fermentation process to create an ideal environment for robust flavonoid and stilbenoid production. Fermentation, as a method, focuses on the cultivation of microorganisms under controlled conditions, and its integration holds tremendous potential for achieving high yields and efficiency. This can be achieved through various strategies, such as manipulating fermentation parameters (temperature, pH, and aeration), optimizing nutrient supplementation, and developing efficient bioreactor designs with improved mixing and oxygen-transfer capabilities [92,93]. In addition, advanced fermentation technologies, such as fed-batch and continuous fermentation, offer more dynamic and controlled growth environments. In feed-batch fermentation, nutrients are added to the fermentation medium in increments, which can help avoid substrate inhibition and improve product yields. On the other hand, continuous fermentation involves the constant addition and removal of media, allowing the microorganism to remain in its exponential growth phase for a prolonged period [94,95].

In conclusion, the most widely reported *C. glutamicum* is a traditional producer for amino acids; however, it is emerging as a promising host to produce flavonoid and stilbenoid for human health and well-being in recent years. *C. glutamicum* was recently shown to produce pinosylvin, resveratrol, piceatannol, naringenin, eriodictyol, kaempferol, and quercetin. Moreover, it also could synthesize the derivatives of flavonoids, such as di-O-methylated pterostilbene, luteolin glucoside, and apigenin glucosides. Although various approaches to metabolic engineering have been applied to produce flavonoid and stilbenoid in *E. coli* and *S. cerevisiae*, the heterologous gene expression of biosynthesis pathway, enhancement of intracellular malonyl-CoA level, and deletion and downregulation of competitive pathways are major approaches to the synthesis of flavonoid and stilbenoid in engineered *C. glutamicum*. Therefore, the yield of flavonoid and stilbenoid is expected to highly improve through the efficient expression of the synthetic pathway and biotechnological strategies. Genomic editing, protein engineering, biosensor-assisted evolution, cofactor regeneration and optimization, and the optimization of fermentable condition can expect a further widening of the product portfolio, as well as the establishment of next-generation techniques with high titers and yields of flavonoids and stilbenoids.

## Figures and Tables

**Table 1:** Summary of flavonoids and stilbenoids production in engineered *C. glutamicium*.

Strains	Genes or Related Gene Cassettes	Products	Substrate/Precusor	Titer (mg/L)	Major Media	Carbon Source	Ref.
*C. glutamicum ATCC 13032*	Anthocyanidin synthase *(ANS)* from *Petunia hybrida*, and 3-O-glucosyltransferase *(3GT)* from *Arabidopsis thaliana;* glucokinase *(GLK),* phosphoglucomutase *(PGM),* UTP–glucose-1-phosphate uridylyltransferase *(GalU1)*	Cyanidin 3-O-glucoside (C3G)	catechin	40	AMM medium	glucose, fructose, or sucrose	[21]
*MB001DelAro^3^*	*STS* from *Arachis hypogaea*, *4CL* from *Petroselinum crispum*	Pinosylvin	cinnamic acid,	121	CGXII medium	glucose	[48]
		Resveratrol	*p*-coumaric acid	158			
		Piceatannol	caffeic acid	56			
	*CHS*, *CHI* from *Petunia hybrida*	Naringenin	*p*-coumaric acid	35			
		Eriodictyol	caffeic acid	37			
*MB001DelAro^4^*	*STS* from *Arachis hypogaea, 4CL* from *Petroselinum crispum, TAL* from *Flavobacterium johnsoniae (TALFj), AroH* of *E. coli*	Resveratrol		59	CGXII medium	glucose	[49]
		Naringenin		32			
*MB001DelAro^4^*	*4CL* from *Petroselinum crispum, STS* from *Arachis hypogaea,* resveratrol-di-Omethyltransferase (*OMT*) from *Vitis vinifera,* maltose-binding protein *MalE* of *E. coli*	di-O-Methylated pterostilbene	*p*-coumaric acid	42	CGXII medium	glucose	[51]
*MB001DelAro^4^*	*4CL* from *Petroselinum crispum, CHS and CHI* from *P. hybrida, F3H* from *Petunia x hybrida, FLS* from *P. deltoides*	Kaempferol	*p*-coumaric acid	23			
		Quercetin	caffeic acid	10			
*MB001DelAro^4^*	*4CL* from *Petroselinum crispum, STS* from *Arachis hypogaea,* 4-hydroxybenzoate: CoA ligase *(HbcL1),* enoyl-CoA hydratase *(EbA5318),* a 3-hydroxyacyl-CoA dehydrogenase *(EbA5320)* and a β-ketothiolase *(EbA5319)* from *“Aromatoleum aromaticum” EbN1;*	Resveratrol	4-hydroxybenzoic acid	5	CGXII medium	glucose	[52]
*MB001DelAro^4^*	*STS Arachis hypogaea, 4CL* from *Petroselinum crispum, TAL* from *Flavobacterium johnsoniae (TALFj), AroH* of *E. coli*	Resveratrol	*p*-coumaric acid	12	CGXII medium	glucose	[45]
*C. glutamicum Nar1_C7*	*4CL* from *Petroselinum crispum, CHS* and *CHI* from *Petunia x hybrida, accBC (cg0802)* and *accD1 (dtsR1, cg0812) of C. glutamicum, ΔfasR, gltA* can be downregulated by exchanging its native promoter to weaker promoter variants citrate synthase *(CS)* coding *gltA (cg0949), ∆sdhCAB (cg0445-47)* operon encoding the succinate dehydrogenase complex (*SDH*)	Naringenin		24	CGXII medium	glucose	[54]
*C. glutamicum* *Res1_C7*	*STS* from *Arachis hypogaea, 4CL* from *Petroselinum crispum, TAL* from *Flavobacterium johnsoniae (TALFj), AroH* of *E. coli*	Resveratrol		112			
*C. glutamicum* *ATCC 13032*	*TAL* from *Rhodotorula glutinis. 4CL* from *Arabidopsis thaliana, Petroselium crispum,* and *Vitis vinifera; CHS* from *Petunia hybrida,* or *Citrus maxima, CHI* of *Citrus maxima* or *Medicago sativa*	Eriodictyol	Tyrosine	14.10	AMM medium	Glucose	[55]
*C. glutamicum* Nor2 C5 mu*fasOBCD1* PO6-*iolT1* ∆*pyc*	*∆cg0344-47, cg0502, cg1226 and cg2625-40*, *4CL* from *Petroselinum crispum* and replacement of the native *gltA* promoter with the *dapA* promoter variant C7 (*PgltA*::*PdapA-C7),* pentaketide chromone synthase from *Aloe arborescens*	Noreugenin	casamino acids	53.32	CGXII medium	Glucose	[56]
*C. glutamicum ATCC 13032*	*YdhE* from *Bacillus licheniformis, galU1* (UDP-glucose pyrophosphorylase), and *pgm* (phosphoglucomutase)	apigenin glucosides	Sorbito		BHI medium	Glucose	[57]
*C. glutamicum* *ATCC 13032*	amylosucrase from *Deinococcus geothermalis*	Luteolin glucoside			BHI medium	Sucrose	[58]

## Data Availability

The data presented in this study are available on request from the corresponding author.
